# Effects of six month personalized endurance training on work ability in middle-aged sedentary women: a secondary analysis of a randomized controlled trial

**DOI:** 10.1186/s12995-020-00261-4

**Published:** 2020-05-06

**Authors:** Hedwig T. Stenner, Julian Eigendorf, Arno Kerling, Momme Kueck, Alexander A. Hanke, Johanna Boyen, Anne-Katrin Nelius, Anette Melk, Dietmar Boethig, Christoph Bara, Andres Hilfiker, Dominik Berliner, Johann Bauersachs, Denise Hilfiker-Kleiner, Jörg Eberhard, Meike Stiesch, Cordula Schippert, Axel Haverich, Uwe Tegtbur, Sven Haufe

**Affiliations:** 1 Institute of Sports Medicine, Hannover Medical School, Carl-Neuberg-Str. 1, 30625 Hannover, Germany; 2 Department of Pediatric Kidney, Liver and Metabolic Diseases, Hannover Medical School, Carl-Neuberg-Str. 1, 30625 Hannover, Germany; 3 Department of Cardiac, Thoracic, Transplantation and Vascular Surgery, Hannover Medical School, Carl-Neuberg-Str. 1, 30625 Hannover, Germany; 4 Department of Cardiology and Angiology, Hannover Medical School, Carl-Neuberg-Str. 1, 30625 Hannover, Germany; 5 Department of Prosthetic Dentistry and Biomedical Material Sciences, Hannover Medical School, Carl-Neuberg-Str. 1, 30625 Hannover, Germany; 6 Department of Obstetrics and Gynecology, Hannover Medical School, Carl-Neuberg-Str. 1, 30625 Hannover, Germany

**Keywords:** Physical activity, Workplace intervention, Work ability index, Subgroups

## Abstract

**Background:**

To test the effects of guided endurance training on work ability in middle-aged female hospital workers of various occupations.

**Methods:**

We randomized 265 healthy, sedentary, middle-aged women (45–65 years) to an endurance training group (EG 210 min/week) or a wait-list control group (CG). At baseline and at 6-month follow-up, we assessed work ability (Work Ability Index [WAI]), physical activity (Freiburger activity questionnaire) and peak oxygen uptake (VO_2peak_) by cardiopulmonary exercise testing. To examine the influence of baseline work ability, participants were divided into poor-moderate (WAI 1, 7–36 points, *n* = 83), good (WAI 2, 37–43 points, *n* = 136) and excellent (WAI 3, 44–49 points, *n* = 46) WAI subgroups.

**Results:**

Cardiorespiratory fitness improved significantly after 6 months in the EG but not in the CG. The WAI total score increased significantly in the EG (38.3 ± 5.0 to 39.8 ± 4.9 points) but not in the CG (39.4 ± 4.7 to 39.3 ± 4.9 points), with a significant difference between groups (*p* < 0.01). In the EG, only the poor-moderate subgroup (WAI 1, 33.0 ± 2.9 to 36.6 ± 4.8 points, *p* < 0.05) increased the WAI total score, with this increase being significantly higher compared to the good (WAI 2, 40.2 ± 2.1 to, 40.4 ± 3.7 points) and excellent (WAI 3, 45.6 ± 1.5 to 45.7 ± 1.8 points) subgroup.

**Conclusions:**

A 6-month guided exercise training intervention significantly increases cardiorespiratory fitness with concomitant improvements in work ability in middle-aged previously sedentary hospital employees. Women with low baseline work ability seem to particularly benefit from the intervention, which implies that similar interventions may be particularly beneficial for this group of individuals.

**Trial registration:**

German Clinical Trails Register Identifier: DRKS00005159. Registered 25 September 2013.

## Background

Work ability is defined as the balance between individual resources and the specific demands of a work task [1]. The demographic changes in most Western societies have been accompanied by the challenge of longer employment. German employees between the ages of 45 and 65 have significantly more inability to work than younger workers [2]. Moreover female workers have more frequent and longer days of illness than men [2]. In 2015, 47,249 million Euros in medical expenses were caused by female employees between 45 and 65 years old, which is a substantially higher amount than that of their younger colleagues (15–45 years 33,582 million Euros) [3].

To assess an individual’s subjective work ability, the Finnish Institute of Occupational Health developed a questionnaire called the Work Ability Index (WAI) [4]. This questionnaire is often used in workplace interventions to measure the effects of interventions [5]. The questionnaire is a kind of early warning system that enables the assessment of the current ability to work and the adoption of measures to maintain and increase the ability to work [6].

A possibility to improve work ability is through regular activity, as shown in previous studies [7, 8]. However, a review [9] concluded that there is insufficient and limited evidence on the effects of interventions for work-related components of aging workers. An even more recent review [5] found that only one-third of randomized studies showed an improvement in work ability through exercise programs. To maintain and possibly increase the ability to work through physical activity in this context, new approaches have to be established. One way is activity promotion during working hours and the other is special focus on eligible employees with a limited ability to work.

The context of female middle-aged employees working in university hospitals may involve unique problems, such as an above average absenteeism rate, working conditions, shift work, time pressure, unsafe employment and a high proportion of physical and psychological pressure [10–13]. Not surprisingly this group of individual’s is prone to exhibit a decreased ability to work and more sick leaves with corresponding costs for the health care system [2, 3]. Therefore, we tested the hypothesis that individualized moderate endurance training partly performed at the workplace improves work ability. We conducted a prospective and randomized controlled study to investigate the effects of a 6-month endurance exercise intervention on work ability in middle-aged sedentary women working at a German university hospital.

## Methods

### Participants and study design

This was a prospective, randomized, parallel-group, and single-blind (assessor blind) study. Participants were recruited through announcements on the institutional intranet, posters, the mail distribution list, and kick-off information events. According to the inclusion criteria, women between 45 and 65 years of age, sedentary lifestyle, low regular physical activity (Freiburger Questionnaire < 20 metabolic equivalents of task (MET)-hours per week) [14], and employed at Hannover Medical School (Lower Saxony, Germany) were included over a period of 18 months (11/2013–05/2015). The cut-off value of 20 MET-hours per week for the classification of low regular physical activity was chosen according to physical activity recommendations from the German Federal Centre for Health Education. The inclusion was distributed over the calendar year to exclude seasonal influences. Exclusion criteria were acute or chronic infections, coronary heart disease, diabetes mellitus, oncological diseases, joint replacements or any surgery within the last 6 weeks, and any condition that precluded the realization of an exercise intervention.

**Fig. 1:**
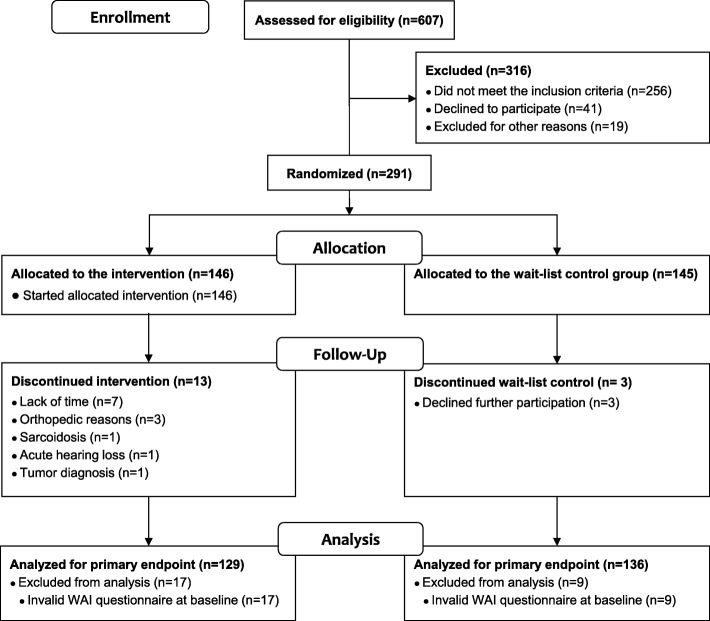
Participant flow chart throughout the study. WAI, Work Ability Index. ^a^More than one reason per participant may have been reported

Throughout the entire period, new participants were randomized 1:1 into an exercise- (EG) and a waiting-control group (CG) using a computer-based list of random numbers generated by an external collaborator (Fig. 1). After their assignment, women in the EG took part in an individualized 6-month exercise intervention. At the beginning and after the 6 months, all women underwent a medical examination by a physician and completed questionnaires. Women in the control group were asked to maintain their current physical activity and dietary habits.

The presented data include a secondary analysis of our study. The primary outcome of this trial, changes in telomere length with 6 months of exercise training, has already been published [15].

This study was carried out in accordance with the Declaration of Helsinki and the current guidelines of good clinical practice. The ethics committee of the Hannover Medical School approved the study (ID: 6428), and written informed consent was obtained from participants before their entry into the study.

### Anthropometrics and body composition

Height was measured using a stadiometer, and body weight was determined using a calibrated scale (seca 764, seca gmbh & co. kg, Hamburg, Germany). The bioelectrical impedance method was used to estimate fat- and fat-free mass (InBody 720, JP Global Markets GmbH, Eschborn, Germany).

### Menstrual status

Pre- or postmenopausal status was set according to the definitions recommended by the World Health Organization (WHO) Scientific Committee in 1980 as the permanent cessation of menstruation resulting from loss of ovarian follicular activity [16]. Menopause for our participants was defined as a 6-month absence of menstruation. Everything else was set as premenopausal.

### Questionnaires

We distributed questionnaires to estimate daily physical activity (Freiburger Physical Activity Questionnaire) and work ability (Work Ability Index [WAI]) [17]. The Freiburger Physical Activity Questionnaire was used to assess the total and exercise-related physical activity of adults, both of which are specified as MET-hours per week. The WAI questionnaire (short form) contains 7 items concerning work, work ability and health: WAI item 1 (current work ability compared with the lifetime best, 0–10 points), WAI item 2 (work ability in relation to the demands of the job, 2–10 points), WAI item 3 (number of current diseases diagnosed by a physician, 1–7 points), WAI item 4 (estimated work impairment due to diseases, 1–6 points), WAI item 5 (sick leave during the past year, 1–5 points), WAI item 6 (own prognosis of work ability 2 years from now, 1–7 points), and WAI item 7 (mental resources, 1–4 points). The sum of these questions results in a total score ranging from 7 to 49 points, with higher values representing greater work ability.

The WAI total score is categorized into four subgroups: 1 = poor (7–27 points), 2 = moderate (28–36 points), 3 = good (37–43 points) and 4 = excellent work ability (44–49 points) [18]. To examine the influence of the level of work ability at baseline, the participants were subgrouped into poor-moderate (WAI 1), good (WAI 2) and excellent (WAI 3) groups. The poor WAI subgroup was combined with the poor-moderate subgroup because of only three cases in the poor subgroup.

### Exercise testing

At baseline and after 6 months, an incremental bicycle exercise test (Ergoline 150P, ergoline GmbH, Bitz, Germany) was performed to measure exercise capacity (maximum workload in Watt) and cardiorespiratory fitness (VO_2peak_). The test started at 20 W, and the workload increased by 10 W every minute until the subjects could not maintain the requested 60 rpm pedal frequency (voluntary exhaustion) or the test was prematurely stopped by the physician due to predefined stopping criteria [19]. We recorded heart rate and blood pressure, and collected capillary blood samples from the earlobe at rest, 1 min after the start and every 3 min during the test to determine blood lactate concentrations. This was followed by a five-minute recovery period at 20 W (MasterScreen CPx, Carefusion, Höchberg, Germany).

### Study intervention: endurance training

The aim of the training intervention was to perform 210 min of endurance training a week (20–60 min units for at least 3 days per week) over 6 months. This duration was based on positive results from our previous study and general recommendations of a minimum of 30 min of physical activity at 5 days per week but most preferable at all days per week [20–22]. We chose endurance exercise at the time of study planning because most evidence pointed on a more pronounced response of endurance training on our primary outcome (telomere length) [23, 24]. The participants in the EG were able to complete part of their training during their working hours (full-time staff 60 min per week and part-time staff 30 min per week) at the in-house health club.

For individualized training, the participants in the EG received heart rate ranges based on the lactate threshold (approx. 60–80% of the estimated max. Heart rate) for their respective activities, such as cycling, rowing and walking. These individual heart rate ranges were based on their prior measured cardiorespiratory fitness and a 30-min constant load test on a bicycle ergometer with 50% of the maximum workload and blood lactate measurements.

To control and document the training heart rates, training content and volume, the participants were equipped with a heart rate monitor, a heart rate belt (PM70, Beurer, Ulm, Germany) and an optional paper or online diary. All participants received an individualized training schedule from an exercise physiologist, which includes advice for specified heart rate ranges, and types and duration of individually performed endurance activities. It was up to the participants whether they completed the endurance activities at home, on commuting to work, in their leisure time or on the exercise machines in the workplace health club. The training schedule also offered the possibility to attend specially created group fitness courses such as Nordic walking, aqua fitness and running with a group size of at least 3 and a maximum of 10 participants. During the whole intervention period, an exercise physiologist supervised and adapted the individual training program.

### Statistical analyses

The normal distribution of data was tested with the Kolmogorov-Smirnov test. Chi-square tests χ^2^ were used to compare sample distributions. Group differences at baseline between the EG and CG were assessed with two-tailed independent t-tests for parametric data or Mann-Whitney U tests for nonparametric data. Data were analyzed by the intention-to-treat (ITT) approach, with the last-observation-carried-forward method for missing data. Within-group differences between baseline and after 6 months were calculated with two-sided dependent t-tests for paired samples for parametric data or Wilcoxon tests for nonparametric data. To analyze the differences between study groups over time, a two-way ANOVA for repeated measures was conducted, where the partial eta-squared (η^2^) was used as the effect size. To compare the group differences between the three WAI subgroups at baseline, a one-way analysis of variance with Bonferroni post hoc tests for parametric data or a Kruskal-Wallis test with post hoc Mann-Whitney U tests for nonparametric data were used. If not otherwise mentioned, all data were presented as the mean ± standard deviation. Significance was accepted as *p* < 0.05. All tests were performed with SPSS Version 25 (SPSS, IBM Corp, Armonk, NY, USA).

## Results

One hundred forty-six women were randomized to the EG and 145 to the CG (Fig. 1). The two study groups were well matched for age, body weight, BMI and cardiorespiratory fitness (Table 1). The largest group of employees was the medical and technical workers (33%), followed by administration (27%), nursing (19%) and physician/scientist (12%). For 9% information is missing, based on the fact that the question about the “job title” was voluntary. Adherence to the goal of 210 min/week of endurance activity per week in the EG was 207 ± 86 min/week (range 10 to 512 min/week). Participants that did not reach the goal of 210 min/week (23%) were also included in the statistical analysis. The analysis of variance showed no significant interaction effect between menstrual status and changes in the WAI total score over time (*p* = 0.067). There was no significant correlation between age and the WAI score at baseline (r = − 0.07, *p* = 0.276).

**Table 1:** Anthropometrics and cardiorespiratory fitness at baseline

Parameter	EG (*n* = 129)	CG (*n* = 136)	*p*-value
Age (yrs.)	53.0 ± 5.0	52.7 ± 4.8	0.588
Body weight (kg)	72.3 ± 13.9	72.6 ± 13.6	0.992
Body mass index (kg/m^2^)	25.7 ± 4.4	26.0 ± 4.6	0.744
Body fat percentage (%)	32.9 ± 7.3	33.2 ± 7.8	0.960
Relative VO_2peak_ (ml/min/kg)	25.4 ± 4.7	25.7 ± 5.1	0.667
Relative workload_max_ (W/kg)	1.98 ± 0.40	2.02 ± 0.43	0.521
Physical activity total score (MET-h/wk)	25.0 ± 18.2	22.2 ± 16.6	0.216
Physical activity sports score (MET-h/wk)	4.8 ± 4.9	5.1 ± 5.2	0.710

*CG* Control group, *EG* Intervention group, *VO*_*2peak*_ Peak oxygen uptake

### Intervention effects

Cardiorespiratory fitness, body weight and body fat percentage improved after 6 months in the EG but not in the CG, with significant differences over time, favoring the EG (Table 2). Furthermore, the physical activity total score and sports-related score increased significantly in the EG compared to the CG (Table 2).

**Table 2:** Anthropometrics, cardiorespiratory fitness and the results of the questionnaires at baseline and after 6 months in the EG and CG

	EG (*n* = 129)		CG (*n* = 136)		Time x group	
Parameter	Baseline	After 6 months	Baseline	After 6 months	*p*-value	*η*^2^
Body weight (kg)	**72.3 ± 13.9**	**71.6 ± 13.5**	72.6 ± 13.6	72.6 ± 13.3	0.014	0.02
Body fat percentage (%)	**32.9 ± 7.3**	**32.2 ± 7.5**	**33.2 ± 7.8**	**33.8 ± 7.4**	< 0.001	0.05
Relative VO_2peak_ (ml/min/kg)	**25.4 ± 4.7**	**27.4 ± 5.2**	25.7 ± 5.1	25.7 ± 5.1	< 0.001	0.09
Relative workload_max_ (W/kg)	**1.98 ± 0.40**	**2.17 ± 0.44**	2.02 ± 0.43	2.02 ± 0.45	< 0.001	0.16
Physical activity total score (MET-h/wk)	**25.0 ± 18.2**	**38.5 ± 23.0**	22.2 ± 16.6	27.8 ± 25.3	0.001	0.04
Physical activity sports score (MET-h/wk)	**4.8 ± 4.9**	**14.3 ± 11.4**	5.1 ± 5.2	8.5 ± 15.3	< 0.001	0.05
**Work ability items**						
WAI item 1 (points)	**7.7 ± 1.4**	**8.1 ± 1.5**	7.9 ± 1.6	7.9 ± 1.6	0.144	0.01
WAI item 2 (points)	**7.8 ± 1.3**	**8.2 ± 1.2**	8.2 ± 1.3	8.1 ± 1.3	0.002	0.04
WAI item 3 (points)	**2.2 ± 1.7**	**1.9 ± 1.6**	2.1 ± 1.7	2.2 ± 1.9	0.047	0.02
WAI item 4 (points)	5.4 ± 0.7	5.4 ± 0.8	5.6 ± 0.6	5.6 ± 0.6	0.715	0.00
WAI item 5 (points)	3.9 ± 0.8	3.9 ± 0.9	3.9 ± 0.8	3.9 ± 0.8	0.586	0.00
WAI item 6 (points)	6.8 ± 1.0	6.7 ± 1.0	6.8 ± 0.7	6.7 ± 1.0	0.309	0.00
WAI item 7 (points)	**3.2 ± 0.8**	**3.3 ± 0.7**	3.2 ± 0.7	3.2 ± 0.7	0.427	0.00

*CG* Control group, *EG* Intervention group, *VO*_*2peak*_ Peak oxygen uptake, *WAI* Work Ability Index. **Bold***p* < 0.05

**Fig. 2:**
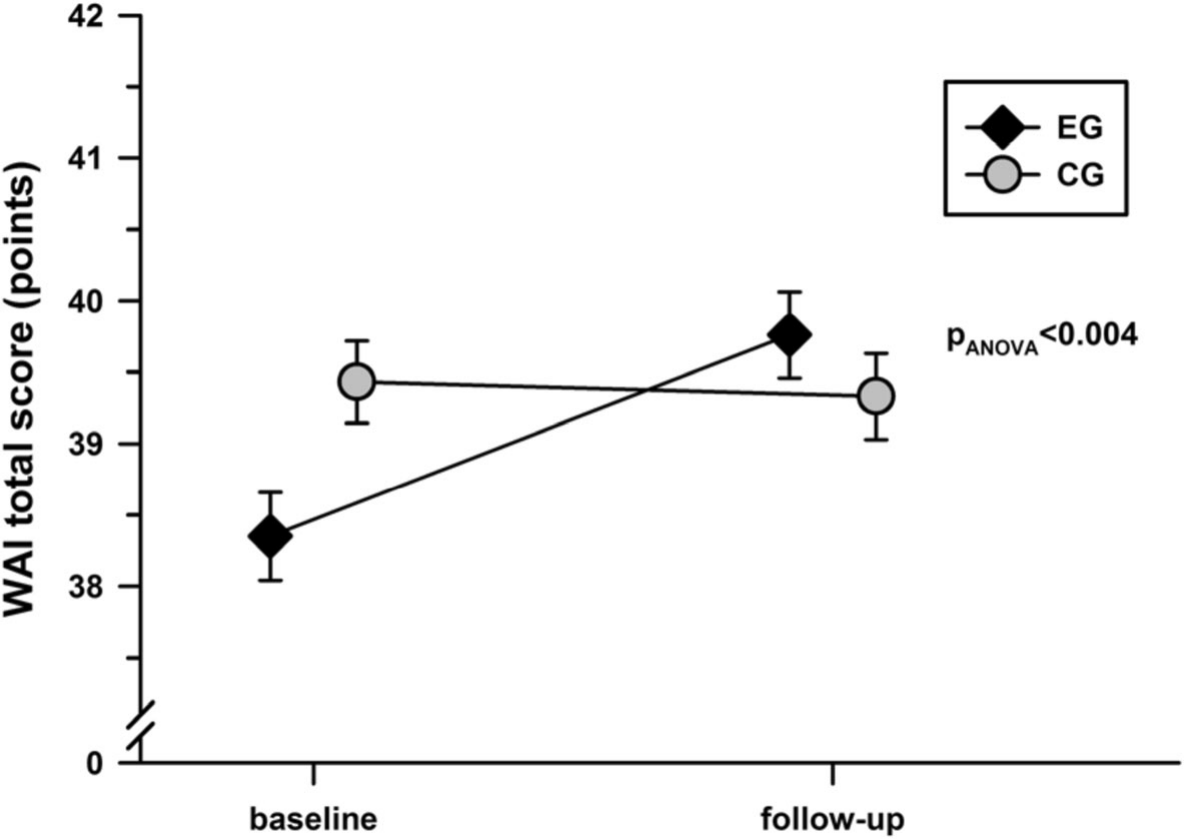
WAI total score in the EG and CG at baseline and after 6 months. There was a significant group x time interaction, as analyzed with a two-way repeated measures ANOVA; there was no significant differences in the WAI total score between EG and CG at baseline (*p* = 0.094); the displayed values are the means ± standard errors. WAI, Work Ability Index

The WAI total score improved significantly in the EG but not in the CG, with a significant difference over time, favoring the EG (Fig. 2). For the WAI subitems, only WAI item 2 and WAI item 3 scores increased significantly (Table 2). A statistically significant greater part of women in EG (33 out of 129) as compared to CG (20 out of 136) were able to improve WAI transferring into a higher WAI group (*p* = 0.032).

### WAI subgroups

Body weight and body mass index were significantly higher, whereas relative VO_2peak_ and relative workload_max_ were lower in the poor-moderate (WAI 1) subgroup compared to the WAI 2 and WAI 3 subgroups (Table 3). There were no differences between the WAI subgroups in age or the physical activity total score and sports-related score.

**Table 3:** Anthropometrics and cardiorespiratory fitness at baseline for the WAI subgroups

Parameter	WAI 1 (*n* = 83)	WAI 2 (*n* = 136)	WAI 3 (*n* = 46)	*p*-value
n (EG/CG)	47/36	64/72	18/28	0.141
Age (yrs.)	53.6 ± 4.6	52.6 ± 4.9	52.3 ± 5.0	0.210
Body weight (kg)	75.9 ± 14.7^a,b^	71.4 ± 13.5^a^	69.6 ± 11.2^b^	0.010
Body mass index (kg/m^2^)	27.1 ± 5.1^a,b^	25.3 ± 4.3^a^	25.0 ± 3.8^b^	0.007
Body fat percentage (%)	34.8 ± 8.3^a^	32.0 ± 7.1^a^	32.8 ± 6.8	0.029
VO_2peak_ (ml/min/kg)	24.0 ± 4.6^a,b^	26.0 ± 4.6^a^	27.2 ± 5.4^b^	< 0.001
Workload_max_ (W/kg)	1.87 ± 0.41^a,b^	2.03 ± 0.38^a^	2.12 ± 0.48^b^	0.002
Physical activity total score (MET-h/wk)	24.0 ± 19.6	24.1 ± 17.3	21.1 ± 13.3	0.655
Physical activity sports score (MET-h/wk)	5.0 ± 4.9	5.0 ± 5.2	4.7 ± 4.9	0.959

*CG* Control group, *EG* Intervention group; ^a^*p* < 0.05 WAI 1 vs WAI 2, ^b^*p* < 0.05 WAI 1 vs WAI 3, *VO*_*2peak*_ Peak oxygen uptake

**Fig. 3:**
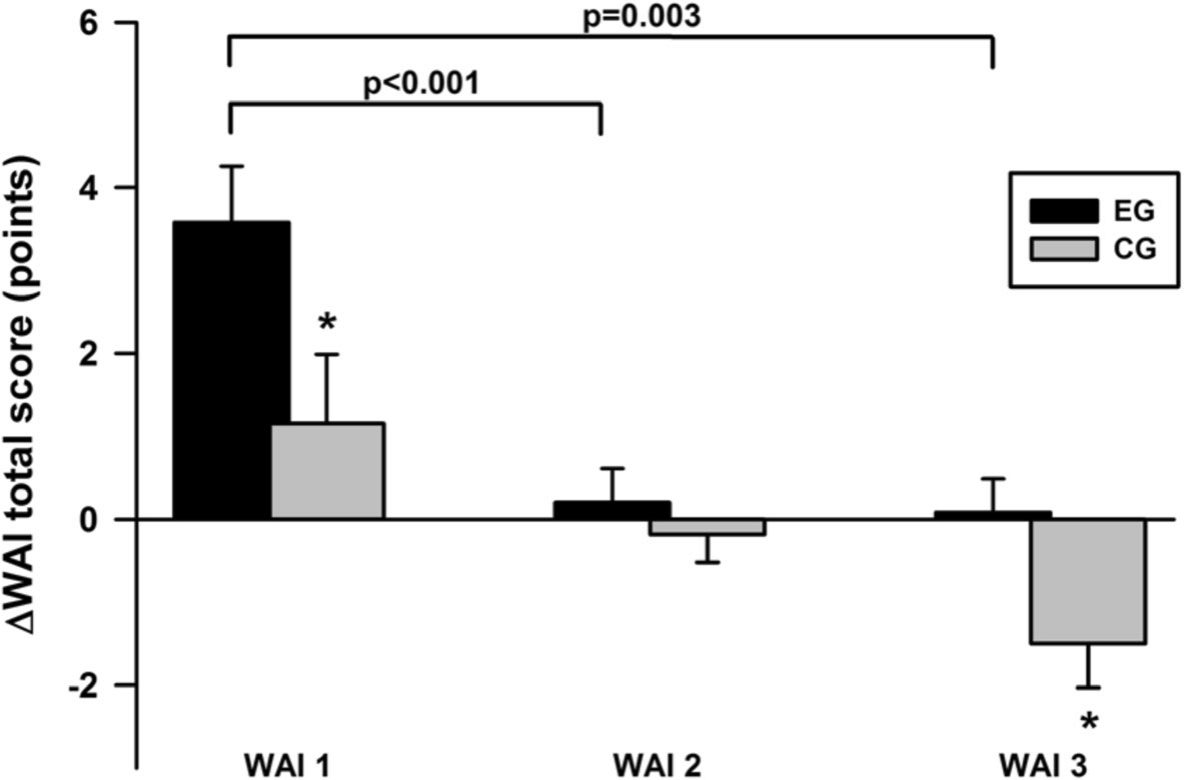
WAI total scores in EG and CG by WAI subgroups from baseline to 6 months. * = *p* < 0.05 for mean differences over time in the WAI between the EG and CG within each WAI subgroup, as analyzed with a two-way repeated measures ANOVA. The *p*-values over the brackets indicate significance of the differences between the WAI subgroups for the WAI changes in the EG, as analyzed with a one-way repeated measures ANOVA and Bonferroni post hoc tests. WAI 1, poor-moderate group; WAI 2, good group; WAI 3, excellent group; WAI, Work Ability Index

Each WAI subgroup had increased VO_2peak_ and workload_max_ after 6 months, without differences between subgroups over time (Table 4). In contrast, body weight and BMI did not change during the intervention (Table 4). For the WAI, a significant time x group (EG vs. CG) interaction was observed for the WAI 1 and 3 subgroups (Fig. 3). In the poor-moderate (WAI 1) subgroup, the exercise training increased the WAI total score significantly more (33.0 ± 2.9 to 36.6 ± 4.8 points) than in the good (WAI 2: 40.2 ± 2.1 to, 40.4 ± 3.7 points) and excellent (WAI 3: 45.6 ± 1.5 to 45.7 ± 1.8 points) WAI subgroups (Fig. 3).

**Table 4:** Mean differences at baseline and after 6 months in anthropometrics, cardiorespiratory fitness and the results of the questionnaires in the EG

Parameter	WAI 1 (*n* = 47)	WAI 2 (*n* = 64)	WAI 3 (*n* = 18)	*p*-value
ΔBody weight (kg)	−1.2 ± 3.4	−0.3 ± 2.7	−1.0 ± 2.3	0.238
ΔBody mass index (kg/m^2^)	− 0.3 ± 1.2	0.0 ± 1.2	− 0.3 ± 0.8	0.285
ΔBody fat percentage (%)	−0.5 ± 2.9	−0.4 ± 3.3	**− 1.7 ± 1.8**	0.155
ΔRelative VO_2peak_ (ml/min/kg)	**2.0 ± 3.3**	**1.9 ± 2.9**	**1.9 ± 3.6**	0.982
ΔRelative Workload_max_ (W/kg)	**0.18 ± 0.20**	**0.20 ± 0.22**	**0.20 ± 0.32**	0.785
**Work ability items**				
ΔWAI total (points)	**3.6 ± 4.7**^**a,b**^	0.2 ± 3.2 ^a^	0.1 ± 1.7^b^	< 0.001
ΔWAI item 1 (points)	**1.0 ± 1.7**^**a,b**^	0.1 ± 1.5^a^	−0.1 ± 1.1^b^	< 0.001
ΔWAI item 2 (points)	**0.8 ± 1.5**^**a**^	0.1 ± 1.0^a^	0.5 ± 1.2	0.030
ΔWAI item 3 (points)	**− 0.7 ± 1.6**^**b**^	−0.2 ± 1.2	0.2 ± 0.6^b^	0.014
ΔWAI item 4 (points)	0.2 ± 0.9^a^	**−0.2 ± 0.6**^**a**^	0.0 ± 0.0	0.015
ΔWAI item 5 (points)	0.1 ± 1.0	0.0 ± 0.8	0.0 ± 0.6	0.952
ΔWAI item 6 (points)	0.1 ± 1.2	−0.1 ± 0.5	0.0 ± 0.0	0.588
ΔWAI item 7 (points)	**0.4 ± 0.8**	0.0 ± 0.7	0.1 ± 0.4	0.042

**Bold***p* < 0.05 baseline vs 6 months, ^a^*p* < 0.05 WAI 1 vs WAI 2, ^b^*p* < 0.05 WAI 1 vs WAI 3. *VO*_*2peak*_ Peak oxygen uptake, *WAI* Work Ability Index

## Discussion

The hypothesis that a 6-month individualized moderate endurance training partly performed at the workplace during working hours would improve work ability has been confirmed. An important finding is that female employees with low baseline work ability seem to particularly benefit from the intervention. This finding supports the classification of individuals according to their work ability before starting an intervention to identify which employees might benefit most from such programs.

Our observations were in line with other studies that have increased the ability to work with exercise training [25–28]. Previous results of a comparable study showed that the control group had a threefold faster reduction of work ability than the intervention group [29]. Therefore, not only have we been able to stop the natural decline but also improve work ability.

We conducted an intervention that offered the possibility to train during the working hours. It has been previously shown that a supervised physical exercise intervention with middle-aged female health care workers is more successful during working hours than during leisure time at home [30]. In addition, physical exercise during work hours is considered to be the most effective form of primary prevention [31]. Given the weekly working time of 39.4 h per week in Germany [32] in the health sector, the workplace should receive special attention. This view is supported by the WHO, which has established the workplace as a priority setting for health promotion [33]. This approach by the WHO has provided a basis for intervention programs performed during the working hours [34]. Not all previous studies have shown positive effects of interventions during working hours on work ability [35, 36]. Reasons for this might be that participants already work physically (e.g., in the case of cleaners, construction workers, and home care workers) or have a high work ability score at baseline, resulting in a ceiling effect. We focused on physically inactive women and observed a high adherence of 98.8%, which was possibly due to their ability to exercise during their working hours and use the workplace health club. This likely facilitated their exercise by decreasing the amount of time and travels required and thus contributed to their incorporation of exercise into the workplace [37]. The personal guidance and regular contact between participants and the supervisor were also likely helpful for strong compliance. Furthermore, problems could be detected early on and solved, thereby maintaining motivation. This approach made it possible to eliminate the two most important barriers for exercise training: lack of time and motivation [38].

For the present study group, it seems reasonable to conclude that an increase in work ability can be slowed by hormonal changes [39, 40]. Therefore, we also investigated the influence of hormonal status and, in contrast to other studies [41, 42], were unable to determine an association between hormonal status and work ability.

In times of demographic change with increasing aging of the working population, maintaining working capacity is of particular interest. In the general population there is a relationship between poor work ability and higher age, as well as resulting earlier retirement [17, 43]. In our group of inactive women we did not observe a significant relation between age and the WAI. However, in that age-group of female employees there might be a particularly increase in physical inactivity and sedentary time [44] which could impact overall health, health-related quality of life and eventually work ability. All three WAI subgroups improved their cardiorespiratory fitness and body composition, with no significant differences between the subgroups. Considering work ability, the poor/moderate (WAI 1) subgroup improved their WAI total score significantly compared to those of the good and excellent subgroups. The good and excellent groups were unable to benefit from the present intervention in terms of the total work ability score. This might indicate the need to determine participants’ current work ability if the goal of endurance training is an improvement in work ability and not fitness alone. Since work ability is easy to estimate it could be a valuable instrument beside established measures like cardiorespiratory fitness or adiposity, in particular when the goal is to increase or maintain an individual’s productivity in relation to job demands or impairment due to diseases. This consideration has already been mentioned in previous studies examining more intensive programs in persons with lower baseline WAI scores [45]. A previous comparable but uncontrolled study observed similar improvements as our intervention after a 12-month program [46]. In this study, the group with a poor/moderate WAI (*n* = 43) increased their scores by approximately 2.9 points after 4 months and by approximately 3.5 points after 8 months, which was consistent with the increase of 3.6 points in our study indicating that 6 months might be sufficient to observe meaningful benefits on work ability.

Considering the individual items, the second WAI item (workability in relation to the demands of the job) and third WAI item (number of current diseases diagnosed by a physician) scores increased significantly in the EG but not in the CG. The women in the EG found themselves more able to cope physically and psychologically with the work requirements and reduces their diseases. This finding is possibly attributable to the positive effect of the increase in performance and body composition throughout the intervention. The women with the lowest baseline work ability again experienced the greatest benefits for these subitems.

The short-term increase in work ability resulting from the intervention is only an intermediate step. For long-term positive outcomes, an intervention should affect all levels of work ability [47]. The basic level (health resources) and the fourth level (work ability) represent the most important effects. However, to be comprehensive, an intervention should include the other levels (competence, values and work) as well. In addition, Lidegaard et al. emphasized that longer interventions may be needed to induce sustained effects on work ability [26]. The investigated age-group is of special interest due to their high labour force participation rate and the related contribution to the gross national product [48]. Furthermore, the demanding work in hospitals is mainly performed by female employees [49]. This situation, together with the decline in physical activity and hormonal changes in women at midlife [39, 44] implicate the need for targeted efforts to promote physical activity across female hospital employees. This indicates the need for further studies with overarching, interdisciplinary and holistic approaches to evaluate long-term effects.

### Limitations

Our study has some limitations. Most of our participants were sedentary due to the predefined inclusion criteria. Therefore, our study is limited to a mainly inactive female working group and cannot be simply extrapolated to other target groups. Due to the nature of our research we have no data on the intervention effect in the long-term, which should be addressed in future studies. Finally, the dropout-rate in the EG was substantially higher as in the CG.

## Conclusions

A 6-month exercise intervention with individualized endurance activities and group fitness courses in middle-aged female hospital workers resulted in positive effects on work ability. For the best outcomes, employees should first be categorized into WAI subgroups to focus on the group with the lowest WAI scores for individualized endurance training. In this context, the support of employees with the weakest work ability could be a beneficial strategy for long-term work ability.

## Data Availability

The datasets used and/or analysed during the current study are available from the corresponding author on reasonable request.

## Abbreviations

CG: Waiting-control group
EG: Exercise-group
VO_2peak_: Cardiorespiratory fitness
WAI: Work Ability Index
WHO: World Health Organization
